# Evaluation of a replication-competent VSV-SIV vaccine candidate

**DOI:** 10.1186/1742-4690-9-S2-P329

**Published:** 2012-09-13

**Authors:** CK Jurgens, G Morrow, C Boggiano, M Panis, J Coleman, R Powell, M Yuan, M Kemelman, N Tamot, M Lopez, A Ouattara, S Iyer, M Backer, K Wright, A Domi, M Chiuchiolo, CR King, M Caulfield, C Parks

**Affiliations:** 1 International AIDS Vaccine Initiative, Brooklyn, NY, USA

## Background

Immunity elicited by live attenuated vaccines confers protection against viral pathogens causing measles, yellow fever, smallpox and others, but this approach is not well suited to HIV vaccine development. Accordingly, to develop a vaccine that incorporates features of a live virus that make it immunogenic without the inherent safety issues associated with attenuated lentiviruses, we are developing replication-competent, recombinant vesicular stomatitis virus (rVSV) vectors for delivery of SIV and HIV vaccines.

## Methods

An rVSV vector was constructed in which the natural glycoprotein (G) was replaced with SIVmac239 Env, and an additional transcription unit was introduced to encode SIVmac239 Gag. The chimeric VSV deltaG-SIV virus vaccine was rescued and evaluated in vitro and in vivo.

## Results

Infection of susceptible cells with this chimeric rVSV-SIV GagEnv virus produced infectious VSV particles containing functional SIV Env, and lentivirus-like particles containing Gag and Env. Serial passage of rVSV-SIV GagEnv vector in CD4+/CCR5+ cells resulted in improved replicative fitness evident by a 2-log increase in infectious virus titers. This adapted virus retained CD4/CCR5 dependence, infected primary rhesus PBMCs and cells isolated from lymph node and duodenum tissues ex vivo. Primary human monocyte-derived dendritic cells (MDDCs) also were susceptible to infection with the rVSV-SIV GagEnv vector. Indian rhesus macaques were immunized with increasing doses of rVSV-SIV GagEnv intramuscularly (IM) to observe reactions to vaccination with this new vector and to quantify immune responses. At the highest dose of 1x10^8^ PFU, no adverse reactions were observed and serum antibody responses against Gag and Env were elicited, which were boosted by IM immunization with Ad5-SIV Gag and Ad5-SIV Env vectors.

## Conclusion

A prototype chimeric VSV-SIV live virus vaccine was developed and evaluated in the NHP model. Chimeric VSV-SIV and VSV-HIV vaccine candidates designed for improved replication and immunogenicity currently are being evaluated in vitro and in vivo.

